# Food intake and blood cholesterol levels of community-based adults with mood disorders

**DOI:** 10.1186/1471-244X-12-10

**Published:** 2012-02-14

**Authors:** Karen M Davison, Bonnie J Kaplan

**Affiliations:** 1 Department of Community Health Sciences, University of Calgary, Calgary, Alberta, Canada; 2 Department of Paediatrics, University of Calgary, and the Alberta Children's Hospital Research Institute, Calgary, Alberta, Canada; 3 IMPART Program, British Columbia Centre of Excellence for Women's Health, Vancouver, British Columbia, Canada

## Abstract

**Background:**

A growing body of literature links nutrition to mood, especially in epidemiological surveys, but there is little information characterizing food intake in people with diagnosed mood disorders.

**Methods:**

Food intake obtained from 3-day food records was evaluated in 97 adults with mood disorders, whose diagnoses were confirmed in structured interviews. Information from a population nutrition survey, national guidelines for nutritional intakes (*Eating Well with Canada's Food Guide*) and North American dietary guidelines (*Dietary Reference Intakes*) was utilized to evaluate the quality of their food intake.

**Results:**

Compared to the regional nutrition survey data and national guidelines, a greater proportion of study participants consumed fewer of the recommended servings of grains (*p *< 0.001) and vegetables and fruits (*p *< 0.05), and less than the lower boundary of the Adequate Macronutrient Distribution Range (AMDR) for α-linolenic acid (*p *< 0.001). The study sample also had greater intakes of high-fat whole grain products (*p *< 0.01), processed meats (*p *< 0.00001), and higher sugar, fat or salty foods (*p *< 0.00001). Of the 1746 total meals and snacks consumed, 39% were from sources outside the home, suggesting a lack of time devoted to meal preparation. Finally, a subsample of 48 participants agreed to have blood tests: 44% had mild hypercholesterolemia (> 5.2 and ≤ 6.2 mmol/L) and 21% had hypercholesterolemia (> 6.2 mmol/L).

**Conclusions:**

Much research has proposed multiple ways in which healthier diets may exert protective effects on mental health. The results of this study suggest that adults with mood disorders could benefit from nutritional interventions to improve diet quality.

## Background

Bipolar disorder and depression are associated with long-lasting disability and significant mortality through suicide, medical illness, and accidental death. As current pharmaceutical treatments have only partial benefit [1], other therapies such as nutritional interventions [2,3] continue to be investigated. Several lines of scientific evidence linking nutrition and mood exist. A substantial number of studies suggest that intake of the essential fatty acids can prevent psychotic disorders [4] or improve mood symptoms [5]. In addition, several nutrition-related concerns have been reported in mood disordered populations [6], such as low income, social isolation, presence of other health problems, drug-nutrient interactions, and suboptimal eating behaviours [7,8]. Finally, recent epidemiologic investigations of dietary intakes suggest the protective effect of better diet quality [9,10]. It appears that there is a compelling reason to examine dietary consumption in people with diagnosed mood disorders; however, to date, no studies have actually compared the intakes of this uniquely vulnerable group to nutritional standards.

## Methods

### Subjects, settings and procedures

The sampling frame for this study consisted of adult (> 18 years) members of the Mood Disorders Association of British Columbia (MDABC) who resided in BC's lower mainland. Using a computerized random number generation analysis tool, the names of 146 randomly selected MDABC adult members were drawn and sent a letter of invitation to participate in a cross-sectional survey of food intake (73 invited in the summer and 73 in the fall/winter). When an individual declined participation, the interviewer attempted to complete a non-response questionnaire asking about lifestyle habits.

When an individual agreed to participate, the research coordinator explained the study over the phone and trained the respondent to complete a three-day food record (reporting three non-consecutive days, two of which were weekdays and one was a weekend day). Participants were also asked if they would provide a blood sample to measure their cholesterol levels.

At the first appointment, participants read and signed the consent form approved by the University of Calgary's Conjoint Health Research Ethics Board. Then they met with a trained clinical interviewer who administered the Structured Clinical Interview for DSM-IV Axis I Disorders [11], the Global Assessment of Functioning (GAF) Scale (GAF) [12] which measured social, occupational, and psychological functioning (scale of 0 to 100), the Hamilton Depression Scale (Ham-D) [13] and, the Young Mania Rating Scale (YMRS) [14]. Those with conditions associated with psychotic symptoms, dementia, thyroid dysfunction, or neuro-degeneration were excluded. Eligible individuals were subsequently interviewed by a registered dietitian who reviewed the participant's food record, administered a validated food frequency questionnaire [15], and asked selected demographic and health related questions. The procedures for collecting dietary data followed that of the British Columbia Nutrition Survey (BCNS), a study of 1823 British Columbians aged 19-84 years. Detailed reports are available at http://www.health.gov.bc.ca/library/publications/year/2004/bcnutritionsurvey.

### Statistical analysis

The software program Food Processor SQL [16], which contains the Canadian Nutrient File [17], was used to analyze the food data. The data were compared with the recommended serving ranges of the national guidelines for Canada, *Eating Well with Canada's Food Guide *(Food Guide) [18], the North American *Dietary Reference Intake*s [19], and data from the BCNS [15,20]. Components of the *Dietary Reference Intakes *that were used were 1) Adequate Macronutrient Distribution Ranges (AMDR): range of intakes for a particular energy source and expressed as a percentage of total energy intake that is associated with reduced risk of chronic disease while providing adequate intakes of essential nutrients, and 2) The Estimated Average Requirement (EAR): a nutrient intake value that is estimated to meet the requirements of half the healthy individuals in a group. If an EAR was not available, Adequate Intakes (AI) were used instead. The AI is a recommended daily intake level based on observed or experimentally determined approximations by a group (or groups) of healthy people [19].

Statistical comparisons between the sample and BCNS as well as analyses within the study group of socio-demographic variables, type of mood disorder and dietary attributes were carried out using Student's t-tests, Mann-Whitney two-sample statistics, binomial tests of two proportions, Pearson's or Spearman's rho correlations, and ANOVA where appropriate.

## Results

### Sample

The overall response rate was 75% (number of participants/number of possibly eligible participants based on the initial phone screening). Of those who declined participation, 44% (11/25) answered the non-response survey: no differences in lifestyle variables (e.g., smoking) were found when compared to the respondents. Sociodemographic characteristics of the sample with comparisons to the BCNS are outlined in Table 1. Slightly more than half of the sample had bipolar disorder (i.e., 59.8% had bipolar disorder and 40.2% had depressive disorder) with relatively high GAF scores (62.7 ± 14.7) suggesting most were only mildly to moderately impaired. The Ham-D scores indicated that 14 (14.4%) were severely depressed, 14 (14.4%) were experiencing moderate depressive symptoms, 44 (45.4%) were mildly depressed, and the remainder were asymptomatic. Based on YMRS scores, 2.6% (all females) were experiencing symptoms of mania. The sample tended to be overweight or obese (67% had a BMI ≥ 25). The main psychiatric medications taken by the sample included various antidepressants (72.9%) and mood stabilizers (52.9%).

**Table 1:** Characteristics of the study (n = 97) and BCNS participants (n = 1823)

Characteristic	Study Sample N (% of total)	BCNS N (% of total)
**Gender:**		

Male	28 (28.9%)**	868 (47.6%)

Female	69 (71.1%)**	955 (52.4%)

**Education level**^1^**:**		

Completed high school or less	21 (21.6%)**	737 (40.8%)

Technical school/some university	46 (47.4%)	810 (44.8%)

University degree	30 (30.9%)***	261 (14.4%)

**Marital status**^2^**:**		

Married or common law	37 (38.1%)***	1178 (65.2%)

Divorced/separated/never married/widowed	27 (27.8%)	358 (19.8%)

Single	33 (34.0%)***	273 (15.1%)

**Smokers**	20 (20.6%)	301 (16.5%)

**p < 0.001

***p < 0.0001

^1^For this variable, total N for BCNS = 1808 and for study sample = 96

^2^For this variable, total N for BCNS = 1806

### Comparison of sample to the regional nutrition survey (BCNS)

Analysis of nutrient intakes and demographic attributes indicated significantly higher intakes of protein [F (3, 93) = 4.40, *p *< 0.05] and fibre [F (3, 93) = 3.07, *p *< 0.05] based on education, and significantly lower intakes for energy (t = -2.19, SE = 195.03, 95% CI = -815.01 to -40.63, *p *< 0.05) and fibre (t = -2.40, SE = 2.87, 95% CI = -12.60 to -1.19, *p *< 0.05) for those who were considered single (i.e., widowed, divorced, separated, or never married). All nutrients were also compared based on age (18 to 34, 35 to 49, and 50+ years for food group analysis and 19 to 30, 31 to 50 and 51 to 70 years for nutrient analysis to be consistent with the standards used). None of the study participants exceeded the age of 70; therefore the subsample of 1320 participants aged 19-70 from the full 1823 BCNS sample was used as the comparison group. Only 19% of participants indicated they were following a therapeutic diet; this subsample had significantly lower carbohydrate intake (255 g ± 103 vs 341 g ± 145; t = 2.40, *p *< 0.05, 95% CI 14.78 to 156.07).

### Comparisons using national guidelines, *Eating Well with Canada's Food Guide*

Eighty-nine (92%) participants claimed to have heard of Canada's Food Guide, but less than one-third (n = 30; 31%) actually used it; there were no significant differences in major nutrient intakes between those who reported that they did and did not use the Food Guide. When compared to the 2007 *Eating Well with Canada's Food Guide *[18] (Table 2), intakes of the study sample within the recommended serving ranges of each of the food groups were significantly lower than the BCNS (*p*'s < 0.05 to 0.0001) with the exception of milk and milk alternatives. Analyses according to age groupings and gender indicated significantly more males between the ages of 18 to 34 and 35 to 49 years ate less than the minimum recommendations for fruits and vegetables (*p *< 0.05) and grains (*p *< 0.001) compared to the BCNS. Significantly fewer females between the ages of 35 to 49 years consumed the recommended 5 to 12 servings of fruits and vegetables per day (*p *< 0.05) compared to the BCNS. There also tended to be more males across all age groups consuming less than 100 g of meat and alternatives per day (range of *p *< 0.05 to *p *< 0.0001). In summary, only 6.2% of the sample consumed the minimum recommended level of servings across all food groups. In order to meet the Food Guide standard, at least 45% of the sample needed to add 2-3 more daily servings of vegetables and fruit; about 15% required 2 extra grain servings daily (30% need 1 extra grain serving); and about half would need to add 1 serving/day of milk and alternatives.

**Table 2:** Food intakes of adults with mood disorders according to *Eating Well with Canada's Food Guide *(CFG) servings and *DRI*s and compared to the BCNS

	Study (n = 97)	BCNS (n = 1320)^1^
**1. Comparisons with CFG servings:**		

**a. Grains (%)**		

< 5 svgs/day	59**	41

5-12 svgs/day	37**	57

> 12 svgs/day	5	2

**b. Vegetables and fruit (%)**		

< 5 svgs/day	75*	65

5-10 svgs/day	23*	32

> 10 svgs/day	2	3

**c. Meat and alternatives (%)**		

< 100 g/day	40*	26

100-300 g/day	37***	66

> 300 g/day	22***	8

**d. Milk and milk alternatives (%)**		

< 2 svgs/day	51***	77

2-4 svgs/day	39***	20

> 4 svgs/day	10***	3

**2. Comparisons with *DRI*s:**		

**a. Carbohydrates**		

i. Grams - median (25^th^; 75^th ^%ile)	305 (215; 405)	

ii. Intake (%) by AMDR^2^		

< 45	27	22

≥ 45 to < 65	65*	76

≥ 65	8	2

**b. Fat**		

i. Grams - median (25^th^; 75^th ^%ile)	90 (64; 123)	

ii. Intake (%) by AMDR^2^		

< 20	3	1

≥ 20 to < 35	46**	74

≥ 35 to < 40	45***	19

≥ 40	5	6

**c. Protein**		

i. Grams of intake per kilogram body weight - median (25^th^; 75^th ^%ile)	1.16 (0.87; 1.53)	

ii. Intake (%) by AMDR^2^		

< 10	4	2

≥ 10 to < 30	94	98

≥ 30	2	0

**d. Saturated fat^3 ^**		

i. Grams - median (25^th^; 75^th ^%ile)	28 (18;38)	

ii. % of total energy intake		

< 5%	5	2

≥ 5 to < 10	38*	51

≥ 10 to < 20	56*	47

≥ 20	1	0

**e. Linoleic acid**		

i. Grams - median (25^th^; 75^th ^%ile)	2.6 (1.6; 3.7)	

ii. Intake (%) by AMDR^2^		

< 5	96**	76

≥ 5 to < 10	3**	24

≥ 10	1	< 1

**f. α-Linolenic acid**		

i. Grams - median (25^th^; 75^th ^%ile)	0.3 (0.1; 0.4)	

ii. Intake (%) by AMDR^2^		

< 0.6	91**	23

≥ 0.6 to < 1.2	7**	70

≥ 1.2	1*	7

*Significant differences between study and BCNS samples at *p *< 0.05

**Significant differences between study and BCNS samples at *p *< 0.001

***Significant differences between study and BCNS samples at *p *< 0.0001

^1^1320 participants from BCNS that were between the ages of 19 to 70 years

^2^Adequate Macronutrient Distribution Range (AMDR) of *Dietary Reference Intakes*

^3^No AMDR is set for saturated fats

*svgs *servings; *g *grams

Note: all statistical results in this table are based on binomial tests of two proportions. The shaded areas represent the recommended levels according to *Eating Well with Canada's Food Guide *or the Acceptable Macronutrient Distribution Ranges (AMDR) of the *Dietary Reference Intakes*

### Comparisons using the North American guidelines: *Dietary Reference Intakes*

**Figure 1:**
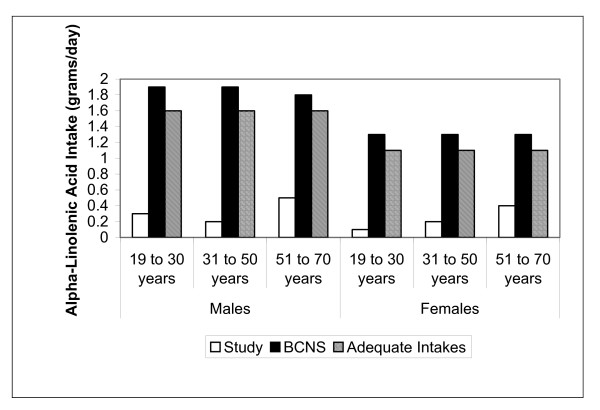
**Median alpha-linolenic acid intakes (grams/day) compared to the BCNS and the *DRIs***.

Median energy intakes (kilocalories) of the study sample were 2520 (25^th ^percentile 1920; 75^th ^percentile 3050); there was slight under-reporting. The study sample had significantly higher total fat intakes (45% vs 19% of the BCNS, *p *< 0.0001) and lower α-linolenic acid intakes (91% vs 23% of BCNS, *p *< 0.001) (Table 2). Median α-linolenic intakes did not meet the AI of 1.6. grams/day for males and 1.1 g/day for females and intakes were consistently lower than the BCNS (Figure 1). About 10% of the sample were below the EAR (0.66 g/kg/day) for protein, which is considered the minimum continuing intake of dietary protein required for body nitrogen equilibrium [19].

Age and gender analysis revealed that a significantly lower proportion of males (12%) between 31 to 50 years had fat intakes that were 30 to 35% of total calories (41% for BCNS). In addition, 2% (all females 51 to 70 years) were consuming less than the EAR for carbohydrates. Median carbohydrate intakes (grams) of females 19 to 30 years (488 vs 257, *p *< 0.05) and 31 to 50 years (326 vs 219, *p *< 0.05) were significantly higher than the BCNS. Only females 19 to 30 years met the AI for fibre. Fibre intakes may be underestimated by about 5 g/day, as inulin and fructo-oligosaccharides were not included [19].

### Description of food intakes by food subgroups and source

Compared to the BCNS, the study sample had significantly greater intakes of less healthy foods such as high-fat whole grain products (e.g., granola cereals), processed meats, and higher sugar, fat or salt foods (range of *p*'*s *< 0.01 to 0.00001) (Table 3). When comparing those who were and were not taking psychiatric medications, there were no differences found for food group, fat and sugar intakes. Participants were also asked about the frequency of meal and snack food intake from sources outside the home based on the three day food record. Of the 1746 total meals and snacks consumed, 11% were from fast food sources, 10% from vending machines or snack bars, 18% from restaurants, take-out or deli. When asked who prepared most of the foods they ate that day, males reported fewer food preparation days (n = 25/84; 29.8%) than females (199/207; 96.1%) (*p *< 0.0001).

**Table 3:** Comparison of portions consumed to the BCNS

Food group	Study % of total portions^a^ (# of total portions)	BCNS % of total portions^a^ (# of total portions)
**1. Grains (total)**	**100 (438)**	**100 (11346)**

Whole***	10.3 (45)	16.6 (1889)

Non-whole - high fat****	66.0 (289)	18.9 (2143)

**2. Fruit (total)**	**100 (152)**	**100 (3954)**

Fruit (deep yellow/orange)	3.9 (6)	4.9 (195)

Fruit (other types)****	39.5 (60)	57.4 (2271)

Fruit juice or nectars****	56.6 (86)	37.6 (1488)

**3. Vegetables (total)**	**100 (346)**	**100 (5397)**

Vegetables (dark green)*	4.9 (17)	9.4 (508)

Vegetables (deep yellow/orange)*	4.6 (16)	9.3 (503)

**4. Milk and milk alternatives (total)**	**100 (607)**	**100 (4104)**

Milk and fortified plant beverages - high fat****	9.1 (55)	29.4 (1208)

Milk products (other) - high fat****	7.9 (48)	25.8 (1060)

**5. Meat**^b ^**(total)**	**100 (27227)**	**100 (252139)**

Meat (beef, poultry, etc.) - high fat****	19.8 (5404)	37.6 (94759)

Fish and shellfish - high fat****	6.5 (1771)	5.7 (14463)

Meat (processed) - high fat****	13.9 (3795)	10.3 (25942)

**6. Meat alternate**^b ^**(total)**	**100 (6305)**	**100 (67781)**

Legumes - high fat***	24.3 (1532)	26.6 (18033)

Nuts and seeds****	5.9 (371)	7.6 (5165)

Eggs*	53.8 (3393)	52.3 (35482)

**7. Other foods**^c ^**(total)**	**100 (161657)**	**100 (3545506)**

Mostly fat, sugar or sodium foods****	75.7 (122421)	3.9 (139494)

Beverages - high calorie^d^****	0.5 (913)	5.4 (189756)

*Significant differences between study and BCNS samples at *p *< 0.05

**Significant differences between study and BCNS samples at *p *< 0.001

***Significant differences between study and BCNS samples at *p *< 0.0001

****Significant differences between study and BCNS samples at *p *< 0.00001

^a^Column totals for each subgroup will not add to 100% because only selected food variables are presented

^b^Expressed as number of 50-gram equivalents consumed: 0-25 grams = 0 serving; 2) 25-49 grams = 1/2 serving; 3) 50-99 grams = 1 serving; 4) 100-300 grams = 2-3 servings, and 5) 301-600 grams = 4-6 servings

^c^Expressed as grams consumed and not portions

^d^Excludes water, coffee, tea, low-calorie soft drinks

### Blood cholesterol levels

Of the 48 participants who agreed to blood tests, 44% had mild hypercholesterolemia (> 5.2 and ≤ 6.2 mmol/L) and 21% had hypercholesterolemia (> 6.2 mmol/L). The prevalence of total cholesterol levels exceeding 5.2 mmol/L in the Canadian population is 41% [21]. Blood cholesterol levels did not differ based on psychiatric medication use, type of mood disorder or BMI status.

## Discussion

The primary aim of this study was to examine the quality of dietary intakes in adults with diagnosed mood disorders using comparisons to national and international nutrition standards as well as regional nutrition survey data. Our results were consistent with others [22] that have revealed many indications of poor diet quality in this population.

The low intakes of grains, vegetables and fruit, and meat and alternatives coupled with the high intakes of foods with excess sugar and fat may compromise mental health status [23,24]. Comparison of carbohydrate (main sources are plants) intakes with the AMDRs showed that more than a quarter of the sample (28%) were below the lower boundary; two participants consumed less than the EAR which is based on the amount needed to produce enough glucose for essential brain activities. About 10% of the sample had intakes of protein less than the EAR, suggesting that important neurotransmitter precursors such as tryptophan may be lacking in the diets of some individuals. Nutrients commonly associated with good mental health include polyunsaturated fatty acids (particularly the omega 3 types), minerals such as zinc, magnesium, and iron, a range of B vitamins particularly folate, and antioxidant vitamins such as C and E most of which are found in diets rich in dark green leafy and orange-coloured vegetables and whole grains. Evidence is accumulating that the combination of polyunsaturated fats, minerals and vitamins may help to relieve the symptoms of some mental illnesses and improve the effectiveness of medication for some conditions [25,26].

Closer examination of the intakes of the major nutrients suggests many implications for negative mental health effects in this population. The high total and saturated fat diets found in this sample have been associated with reduced hippocampal levels of neurotrophic factor, a crucial modulator of synaptic plasticity [27], which can induce cognitive dysfunction [28]. Low intakes of omega-3 fatty acids impair astrocyte-mediated vascular coupling that contributes to reduced gray matter volume in the prefrontal cortex [29] and research has suggested that lipid profiles comprising a low docosahexaenoic acid percentage and omega-3 proportions predicted risk of suicidal behaviour among depressed patients over a 2-year period [30]. The high proportion of participants with hypercholesterolemia (further suggesting excess fat intakes) also has mental health implications. Studies of people with elevated blood cholesterol levels have shown that global severity of psychological symptoms is worsened with high fat, low-complex carbohydrate diets [31]. Lipid-lowering medications are standard treatment for hypercholesterolemia, however, these drugs can form complexes with lipoproteins, alter the medication's pharmacokinetics and lead to deterioration of mental symptoms [32]; dietary interventions do not present these risks.

The limitations of this study include biases related to recall, sample selection (i.e., participants were drawn mainly from an urban sample of a non-profit network), and misclassification (i.e., with AMDRs). Males were under-represented, which may limit generalizability. The samples compared (i.e., study versus BCNS) did differ on some variables (e.g., income levels) that can affect food choice, however both samples were drawn based on random selection. Finally, this cross-sectional study cannot determine the temporal sequence of disease and nutrient intake.

One of the study's strengths is that it provided quantified comparisons of nutrient intakes to the general local population, which have never been reported previously for mood disorders. This investigation also determined the prevalence of various nutrition-related factors in a mood disorder sample and is therefore useful for future hypothesis generation and planning of health services.

Dietary intakes in this sample of adults with mood disorders tended to consist of a high proportion of foods associated with neuronal impairment (e.g., high fat, high sugar) and limited dietary components associated with neuro-protection (e.g., fibre, antioxidants). People with mood disorders have a greater frequency of poor diet for several reasons such as the occurrence of depressive episodes that exacerbate a sedentary lifestyle associated with lack of exercise, weight gain, and cardiovascular disease and diabetes risk, or manic episodes that may be associated with treatment non-adherence. Other factors such as food insecurity, co-existing medical problems, or substance use may also explain the association between mood disorders and poor nutrition status. The negative impact of poor dietary intake in individuals with mood disorders may be exacerbated by the fact that health providers are unlikely to discuss diet habits with them, according to patient report [33].

## Conclusions

Based on these findings, it is evident that individuals with mood disorders would benefit from diet-specific interventions that go beyond familiarization with *Canada's Food Guide *to optimize health. However, there is lack of research indicating what types of nutrition-related programs would be most effective for individuals with serious mental disorders. Future directions in the area of diet in mental disorders should focus on interventions that include appropriate modification of the major nutrients and that increase foods offering neuroprotection and the investigation of their outcomes.

## Abbreviations

AMDR: Adequate macronutrient distribution ranges; EARs: Estimated average requirements; BCNS: British Columbia Nutrition Survey; AI: Adequate Intake.

## Competing interests

The authors declare that they have no competing interests.

## Authors' contributions

KMD carried out this study as part of her requirement for a PhD in the Faculty of Medicine, University of Calgary, under the supervision of BJK, and is currently a postdoctoral research fellow with the IMPART Program at the British Columbia Centre of Excellence for Women's Health. BJK is a Professor in the Faculty of Medicine at the University of Calgary who studies nutrition in relation to mental development and function.

## Pre-publication history

The pre-publication history for this paper can be accessed here:

http://www.biomedcentral.com/1471-244X/12/10/prepub
